# Rheological Performance of Magnetorheological Grease with Embedded Graphite Additives

**DOI:** 10.3390/ma14175091

**Published:** 2021-09-06

**Authors:** Nur Alyaa Mohd Nasir, Nurhazimah Nazmi, Norzilawati Mohamad, Ubaidillah Ubaidillah, Nur Azmah Nordin, Saiful Amri Mazlan, Siti Aishah Abdul Aziz, Muhammad Kashfi Shabdin, Nurul Azhani Yunus

**Affiliations:** 1 Engineering Materials and Structures (eMast) iKohza, Malaysia-Japan International Institute of Technology (MJIIT), Universiti Teknologi Malaysia, Jalan Sultan Yahya Petra, Kuala Lumpur 54100, Malaysia; alyaanasir0@gmail.com (N.A.M.N.); nurazmah.nordin@utm.my (N.A.N.); amri.kl@utm.my (S.A.M.); aishah118@gmail.com (S.A.A.A.); 2 Faculty of Engineering, Universiti Malaysia Sabah, Jalan UMS, Kota Kinabalu 88400, Malaysia; norzilawati@ums.edu.my; 3 Mechanical Engineering Department, Universitas Sebelas Maret, J1. Ir. Sutami 36A, Kentingan, Sukarta 57126, Indonesia; ubaidillah_ft@staff.uns.ac.id; 4 Department of Physics, Faculty of Science, Universiti Putra Malaysia, Serdang 43400, Malaysia; kashfi.shabdin@upm.edu.my; 5 Mechanical Engineering Department, Universiti Teknologi PETRONAS, Seri Iskandar 32610, Malaysia

**Keywords:** magnetorheological grease, graphite, non-magnetic particles, rheological properties

## Abstract

The use of highly viscous grease as a medium in magnetorheological grease (MRG) provides the benefit of avoiding sedimentation from occurring. However, it limits the expansion of yield stress in the on-state condition, thus reducing the application performance during operation. Therefore, in this study, the improvement in the rheological properties of MRG was investigated through the introduction of graphite as an additive. MRG with 10 wt % graphite (GMRG) was fabricated, and its properties were compared to a reference MRG sample. The microstructure of GMRG was characterized using an environmental scanning electron microscope (ESEM). The rheological properties of both samples, including apparent viscosity, yield stress, and viscoelasticity, were examined using a shear rheometer in rotational and oscillatory modes. The results demonstrated a slight increase in the apparent viscosity in GMRG and a significant improvement in yield stress by 38.8% at 3 A with growth about 32.7% higher compared to MRG from 0 to 3 A. An expansion of the linear viscoelastic region (LVE) from 0.01% to 0.1% was observed for the GMRG, credited to the domination of the elastic properties on the sample. These obtained results were confirmed based on ESEM, which described the contribution of graphite to constructing a more stable chain structure in the GMRG. In conclusion, the findings highlight the influence of the addition of graphite on improving the rheological properties of MRG. Hence, the addition of graphite in MRG shows the potential to be applied in many applications in the near future.

## 1. Introduction

Magnetorheological (MR) materials are categorized as smart materials, as their rheological properties can be manipulated with the application of a magnetic field. Magnetorheological fluids (MRFs) are a kind of MR material that have been widely applied in various applications such as in shock absorber, actuator, clutches, dampers, and brakes due to their outstanding properties [1,2]. However, MRFs exhibit sedimentation problems after some time because of the density mismatch between the magnetic particle and carrier fluid [3]. Therefore, magnetorheological grease (MRG) was invented to overcome this problem. MRG was created by Rankin et al. [4]; it is capable of solving the sedimentation problem that arose in MRFs and the low MR effect in magnetorheological elastomers (MREs). The use of grease, which is a non-Newtonian fluid, as a medium in MRG enables the magnetic particles to be suspended against the gravitational force; thus, the sedimentation of particles can be eliminated [3,5]. Moreover, grease is classified as an intermediate state between fluid and solid, providing a certain freedom of movement to the magnetic particles to form columnar chains under the influence of a magnetic field [6]. Consequently, a higher MR effect of 952.38% was recorded for MRG compared to the 71.7% of solid-like-state MRE [7,8]. Additionally, compared with MRF, MRG does not require additional sealing to prevent any leakages of the devices as MRG has a self-sealing property due to its thick viscosity. This property can maintain the stability of the equipment for long-term usage, thus reducing the manufacturing cost [9]. The merits mentioned above have led to MRG becoming a potential candidate for application in engineering as seismic dampers, brakes, and clutches [1]. However, the use of grease as a medium for MR material resulted in the MRG experiencing high off-state viscosity, thereby limiting the expansion of yield stress in the on-state condition. This has led to MRG exhibiting poor performance in indicators such as torque output [10].

The rheological properties of MRG can be improved by the use of additives. Kim et al. [11] investigated the influence of kerosene oil as an additive on the rheological properties of MRG. They discovered that the apparent viscosity of MRG was reduced by the addition of 5 wt % kerosene, which indicated a better dispersion of carbonyl iron particles (CIPs) in the grease medium. However, the dynamic yield stress and viscoelastic properties of MRG also decreased simultaneously. Their findings are consistent with the those reported by Mohamad et al. [12], who used and compared three different types of dilution oils, namely kerosene oil, castor oil, and hydraulic oil, in MRG. Even though they discovered that the usage of these types of dilution oils was able to reduce the off-state viscosity of the MRG, the dynamic yield stress of the MRG also decreased. In other words, the addition of dilution oils to MRG may lower its apparent viscosity; however, it would make the CIPs less attached to the grease medium, leading to a slipping effect under the influence of shear stress, which would cause a drop in the resultant yield stress. Consequently, performance would decrease, especially under the influence of low magnetic field strengths. Recently, Wang et al. [13] optimized a method to fabricate MRG through an ANOVA of many parameters such as CIP fraction and size, and silicone oil viscosity. They found that the optimum yield stress can be obtained by manipulating the fraction of CIPs and silicone oil viscosity, but the influence of CIP size was negligible. However, they noted that as the used silicone oil viscosity was higher, a drop in the yield stress of MRG was observed at a higher magnetic field strength. The reasons for this finding are possibly the use of high-viscosity silicone oil up to 1000 m^2^s^−1^ in MRG, contributing to the rise in MRG’s apparent viscosity, which finally restricted the alignment of CIPs in the medium under the influence of magnetic fields.

Apart from using different types of dilution oil as an MRG additive, several studies incorporated solid-type additives to enhance the rheological properties of MRG. For example, Park et al. [3] revealed that adding nanoparticles as an additive (CrO_2_ in MRG) helped improve the stability of MRG, resulting from the steric repulsion effect between the CIPs and CrO_2_. However, the dynamic yield stress of MRG showed an insignificant improvement. Mohamad et al. [14] introduced another type of nanoparticle additive in MRG, namely, super-paramagnetic γ-Fe_2_O_3_. The addition of 1 wt % additive was capable of lowering the off-state viscosity and increased the on-state viscosity of MRG. The result reflects the effect of nano-sized particles that fill the voids between the CIPs under the influence of magnetic fields, thus contributing to the formation of stronger chain-like structures inside the medium. Later, Tarmizi et al. [15] used a micron-sized additive, cobalt ferrite (CoFe_2_O_4_), which further lowered the off-state viscosity of MRG by up to 86% at 1 wt %. They reported the highest yield stress obtained, about 12 kPa, with the incorporation of 5 wt % of CoFe_2_O_4_ at 0.64 T of the applied magnetic field. However, it was noted that the range of expansion of yield stress in MRG with the incorporation of 5 wt % CoFe_2_O_4_ was considered low, ranging from 0.8 to 12 kPa by increasing magnetic field from 0 to 0.64 T. Apparently, this yield stress range limits the material’s wider application.

In addition to using solid magnetic additives to improve the rheological properties of MR material, the incorporation of non-magnetic, carbon-based additives such as graphite could alternately enhance the rheological performance of MR materials [16,17,18,19]. Graphite possesses excellent properties, such as good thermal and electrical conductivities, mechanical properties, chemically inertness, and low density, so it is capable of maintaining the material’s existing mechanical properties. The incorporation of graphite into MRG may induce electrical properties that make MRG a dual-behavior MR material. Moreover, graphite can be classified as an economical additive due to its high availability and low-cost production [20]. An experimental study conducted by Tian et al. [16] showed that the initial mechanical properties of MRE are improved by the addition of 20 wt % graphite. Then, a noticeable improvement in the MR effect up to 60% in the field-dependent modulus of MRE was confirmed by Shabdin et al. [17] with the addition of 33 wt % graphite. In their study, the MR effect was improved by 176% compared to the previous study [16].

Other MR materials such as MR plastomer (MRP) and MRF have also benefited from the use of graphite as an additive. The addition of 15 wt % graphite in MRP increased the saturated storage modulus by 0.8 MPa compared to pure MRP, and the viscosity was remarkably improved due to the strengthening effect exhibited by graphite [18]. In another study performed on MRF by Thakur [19], high on-state viscosity and shear stress values were obtained by increasing the weight percentage of graphite flakes up to 3%. The authors stated that this was caused by the contribution of graphite to improving the formation of columnar chain structure by filling the empty gaps between the CIPs to form stronger structures. As a result, the yield stress elevated.

Therefore, it can be concluded that the incorporation of graphite improves the rheological properties of MR materials, and it is expected that it would improve the rheological properties of MRG as well. To date, the effects of the incorporation of graphite in MRG on the rheological properties in terms of apparent viscosity, shear stress, yield stress, and viscoelastic properties has not yet been deeply investigated. Hypothetically, it is expected that a wider range of yield stress can be achieved along with increases in the applied magnetic field. Therefore, in this study, a carbon-based additive, graphite, was used in the fabrication of a new MRG called GMRG. Although the off-state viscosity of MRG is presumed to slightly increase with the addition of graphite powder, the field-dependent yield stress of MRG can be enhanced due to the strengthening effect of the graphite. The samples were tested in terms of rotational and oscillatory rheological behavior using a commercial rheometer with a parallel-plate measuring cell. The performance of GMRG, including yield stress and viscoelastic properties, was compared and discussed with a reference sample (MRG, without graphite).

## 2. Methodology

### 2.1. Sample Preparation

The samples consisted of soft magnetic CIPs (OM grade, BASF, Ludwigshafen, Germany) with an average size and density of 5 μm and 7.874 g/cm^3^, respectively, dispersed in commercial lithium grease (NPC-Highrex HD-3 Grease, Nippon Kyu Ltd., Tokyo, Japan). The density and viscosity of the used grease were 0.92 g/cm^3^ and 0.207, Pa s, respectively. The additive used in this study was irregular-shape graphite (R&M Chemicals, EverGreen Engineering and Resources Co., Kuala Lumpur, Malaysia) with an average size of 16 μm and density of 1.8 g/cm^3^. In general, MRG was fabricated by direct-mixing of CIPs and grease, and GMRG was fabricated with the addition of graphite. Specific compositions for both MRG and GMRG samples are shown in Table 1. Initially, the samples were prepared by stirring the grease for 5 min to open the fibrous structures. Subsequently, either CIPs and graphite (GMRG) or CIPs only (MRG) were added into the grease and stirred at 300 rpm continuously for 2 h using a mechanical stirrer until a homogenized mixture was attained. All procedures were conducted at room temperature.

### 2.2. Sample Characterization

The microstructure of GMRG was examined via an environmental scanning electron microscope (ESEM; Quanta 450 FEG, FEI) to observe the distribution of the CIPs and graphite in the grease medium. A thin layer of platinum was used to coat the sample prior the analysis to avoid the electron charging of the samples. The rheological properties of MRG were characterized using a parallel-plate rheometer (Anton Paar, Physica, MCR 302) under rotational and oscillatory modes. The shear rheometer was equipped with an MR device (MRD70/1T) to generate magnetic fields in the rheometer that varied from 0 to 0.603 T by adjusting the input current from 0 to 3 A. The values of 0, 1, 2, and 3 A correspond to 0, 0.212, 0.418, and 0.603 T, respectively. For each measurement, 1 mL of sample was filled on the parallel plate with a diameter of 20 mm and a constant gap of 1 mm. The apparent viscosity and shear stress of MRG and GMRG were determined by manipulating the shear rate from 0.01 to 100 s^−1^ under continuous mode. The elasticity and energy dissipation of MRG and GMRG were evaluated through sweep strain tests. The strain was varied from 0.001% to 10% at a constant frequency of 1 Hz. All experiments were conducted at room temperature (25 °C). The temperature of the rheometer was set and controlled by a Viscotherm VT2 to maintain the desired temperature throughout the experiment.

## 3. Results

### 3.1. ESEM Characterization

**Figure 1:**
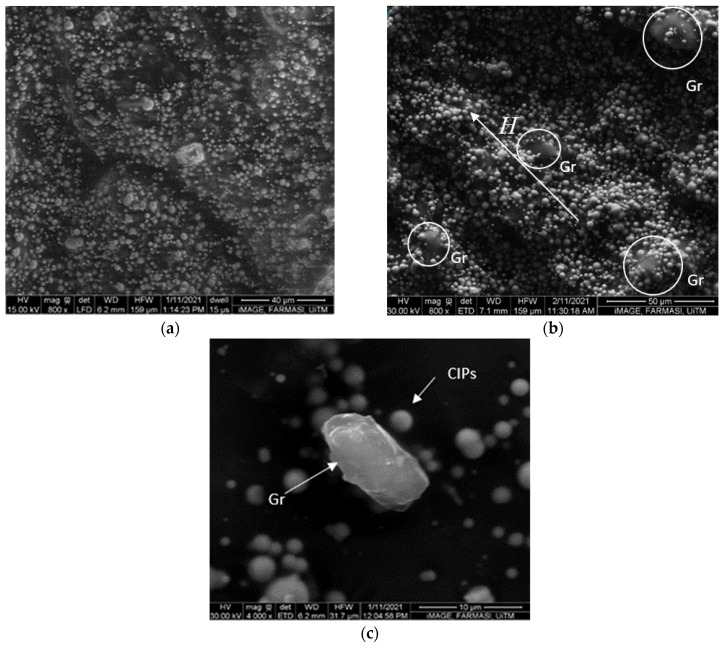
ESEM images of GMRG samples (**a**) in the absence of a magnetic field and (**b**) pre-treated with a magnetic field of 0.1 T at magnification of 800×; (**c**) an enlarged view of CIPs and graphite in the absence of a magnetic field at a magnification of 4000×.

The ESEM images of the GMRG sample are illustrated in Figure 1. Figure 1a displays an image of the sample without the influence of a magnetic field at magnification of 800× with an acceleration voltage of 15 kV; Figure 1b depicts the sample pre-treated with a magnetic field of 0.1 T at magnification of 800× with an acceleration voltage of 30 kV; Figure 1c shows enlarged views of the microstructure of the CIPs and graphite in the GMRG sample at a higher magnification of 4000× with a similar acceleration voltage of 30 kV.

Generally, the CIPs and graphite were randomly distributed in the grease medium in the absence of a magnetic field (Figure 1a). Then, under the influence of a magnetic field, the CIPs were attracted to each other through dipole–dipole force and started to align in the direction of the applied magnetic field. Simultaneously, graphite particles vibrated together with magnetically influenced CIPs, thus resulting in the aligning process forming a stronger columnar chain structure (Figure 1b). Moreover, Figure 1c confirms that the used CIPs and graphite have spherical and irregular shapes, respectively. However, some graphite was captured due to the small weight percentage of graphite compared to CIPs. We also observed that graphite showed a good dispersion in the grease matrix without the existence of any agglomeration.

**Figure 2:**
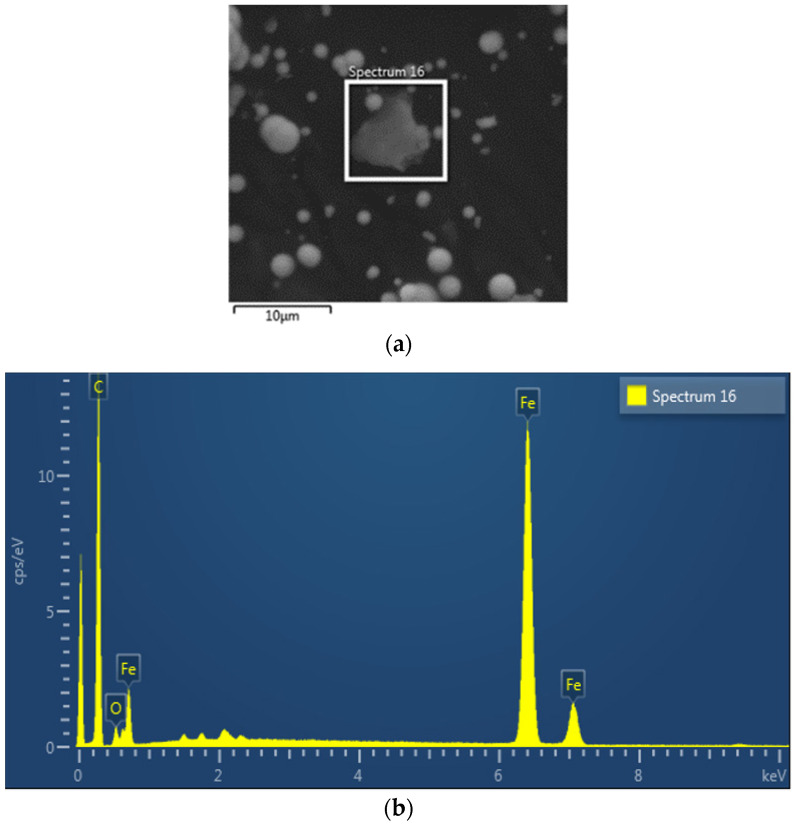
(**a**) Selected microstructure area and (**b**) EDX graph for the GMRG sample.

In terms of the ESEM results, as illustrated in Figure 2b, an EDX analysis was performed at the selected area, spectrum 16 (Figure 2a), to confirm the elemental composition of the GMRG sample.

Table 2 present the GMRG elemental composition from the EDX analysis. It shows that, carbon exhibited the largest proportion of around 75.34 wt %, followed by iron and oxygen with proportions of 17.64 wt % and 7.02 wt %, respectively. As elemental carbon in the two-dimensional structure of graphite has the lowest energy state, it is the most stable form of carbon in standard conditions (ambient temperature and pressure); therefore, it reflects the constituent with the highest proportion in the result [21]. Additionally, a large proportion of carbon occurred due to the hydrocarbon chain from the grease matrix, which also contributed to the proportion of oxygen. This was due to the type of grease utilized in this study being based on a lithium-12-hydrostearate thickener, which consists of hydroxyl (-OH) and carboxylate (-COO^−^) functional groups [22,23]. Furthermore, the second-highest constituent of Fe was contributed from the CIPs that are normally composed of pure iron.

### 3.2. Effect of Graphite on MRG under Rotational Mode

**Figure 3:**
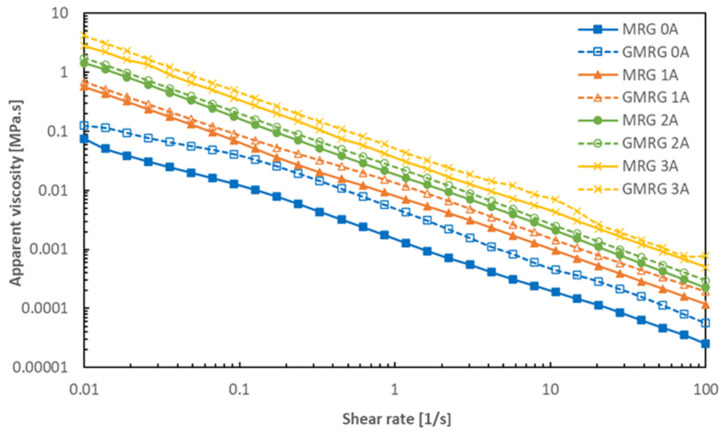
Apparent viscosity of MRG and GMRG in a shear rate range from 0.01 to 100 s^−1^ under different applied currents.

Figure 3 presents the apparent viscosity of MRG and GMRG as a function of shear rate at various magnetic field strengths.

Both samples, MRG and GMRG, experienced a shear-thinning phenomenon in the absence and presence of magnetic fields [24,25]. The apparent viscosity of both samples decreased with increasing shear rate. This phenomenon is related to the destruction of CIP alignment as well as the alteration in the grease medium under the influence of high shear force [26]. Furthermore, the apparent viscosity of both samples increased as the applied current increased from 0 to 3 A, corresponding to the formation of a strong columnar chain between CIPs along the direction of the applied magnetic field [7].

We also observed that GMRG exhibited a higher apparent viscosity at each magnetic field strength compared to MRG with increasing shear rate. For example, under the off-state condition, the value of the initial apparent viscosity of GMRG increased about 0.049 MPa.s compared to the MRG sample. In the absence of a magnetic field, the apparent viscosity of MRG was only dependent on the fibrous structure of the grease matrix [1,24]. However, the addition of graphite to GMRG produced a thickening effect on the grease matrix [27], as the use of graphite increased the amount of solid content in the GMRG sample that also contained CIPs. Consequently, a larger number of particles interacted with the polymer; as a result, the flow resistance of the medium increased [28]. Even so, the increase in the apparent viscosity of GMRG was still low because the density of used graphite is much lower compared to CIPs—about four times lower.

In the presence of a magnetic field, GMRG showed a slight increase in apparent viscosity compared to MRG. From the result obtained, it can be proven that although graphite is a non-magnetic particle, it can be involved in the process of alignment with CIPs to form stronger structures in the presence of a magnetic field. The result is in agreement with that of Zhang et al. [29]. Furthermore, this finding is consistent with the previous studies that used graphite to improve the rheological properties of MR material [17,18]. However, GMRG exhibited unstable apparent viscosity under an applied current of 3 A due to the formation of a thicker structure, which was attributed to the graphite particles with increasing magnetic field strength. Thus, this formation might contribute to the slip of the parallel-plate rheometer at a high shear rate [30].

**Figure 4:**
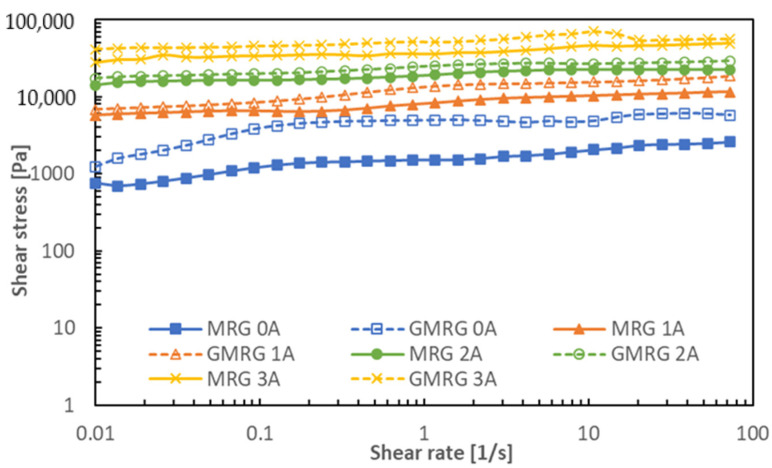
Comparison of shear stress between MRG and GMRG at various magnetic field strengths.

The shear stress assessment with a shear rate range of 0.01 to 100 s^−1^ at various applied magnetic field strengths is provided in Figure 4.

A fluctuating shear stress trend was found for GMRG in the off-state condition. The increasing solid content in GMRG caused extra collisions between the particles and the medium under the influence of shear force. Furthermore, graphite’s larger size and irregular shape led to disorderly motion in the grease medium. Nonetheless, the shear stress trend of GMRG was more stable with the application of the magnetic field compared to the others. Moreover, at low shear rate, we observed that both samples showed a linear increase in shear stress. This occurred because the CIPs in the medium were not stable at low shear rate as the formation of columnar chain structure was initiated but hindered due to shear force. However, the shear stress at higher shear rates showed a stable trend due to the strong dipole–dipole interaction between the CIPs, perpendicular to the direction of shear flow. It was found that the shear stress of MR suspension manageably improves with the implementation of a high shear rate [31].

Additionally, GMRG demonstrated a high shear stress compared to MRG in the off-state condition. This result corresponds to the additional particles in GMRG that were attributed to CIPs and graphite, which participated in the process of shear. The addition of graphite contributed to increasing the number of particle–particle and particle–medium interactions; however, it resulted in increased friction between the particles and the medium. Subsequently, the interactions induced the flow resistance of the medium and, as a consequence, the shear stress of the GMRG sample increased [28]. Interestingly, the effect of graphite on shear stress was also obvious in the presence of a magnetic field. We observed that GMRG exhibited higher shear stress compared to MRG at all magnetic field strengths. Notably, as the applied magnetic field strength increased, a thicker columnar chain structure between CIPs formed. Simultaneously, the existence of irregular-shaped graphite with a high surface contact area promoted the agglomeration of CIPs around their surface [32]. Subsequently, as the density of graphite is much lower than that of CIP, graphite was easily being escorted by CIPs in the alignment process. Based on the findings, graphite particles were indirectly involved in the formation of the columnar chain structure to produce a more robust and stable structure, reflected by the result of the increase in the shear stress of GMRG.

**Figure 5:**
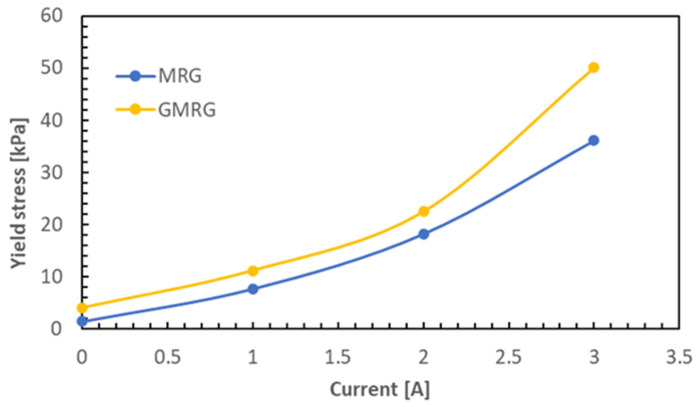
Yield stress of MRG and GMRG under various applied currents from 0 to 3 A.

From the relationship between shear stress and shear rate, the yield stress for both samples was acquired through extrapolation from the zero shear rate. Figure 5 shows the yield stress of MRG and GMRG as a function of applied current from 0 to 3 A.

A linear trend of enhancement in the yield stress was observed for both MRG and GMRG samples with increasing magnetic field. The yield stress of MRG showed an increase from 1.375 to 36.051 kPa, and 4.0167 to 50.048 kPa for GMRG, with increasing the applied current from 0 to 3 A. The increase in yield stress resulted from the formation of stable chain structures within the medium with increasing magnetic field strength. This is because the stiffness of both samples increased with the magnetic field strength, thus hindering the free movement of CIPs within the medium. The results showed that the growth of yield stress in GMRG was about 32.7% higher than that of MRG from 0 to 3 A, and 311% higher compared to the findings reported in [15]. Hence, we proved that graphite can help to expand the range of yield stress of MRG due to its strengthening effect.

As shown in Figure 5, the addition of graphite to GMRG improved in the yield stress at all applied currents. We noted that the yield stress of MRG increased by about 38.8% with the addition of 10 wt % graphite at 3 A. Furthermore, the yield stress of GMRG showed a dramatic increase starting from 2 to 3 A, demonstrating a different trend compared to MRG. With a further increase in the magnetic field strength, the CIPs in the medium that already formed thicker and stronger chain structures reached a stable structure formation. Consequently, a higher force was required to break the new structure, which directly caused a sudden rise in the yield stress of GMRG.

### 3.3. Effect of Graphite on MRG under Oscillatory Mode

**Figure 6:**
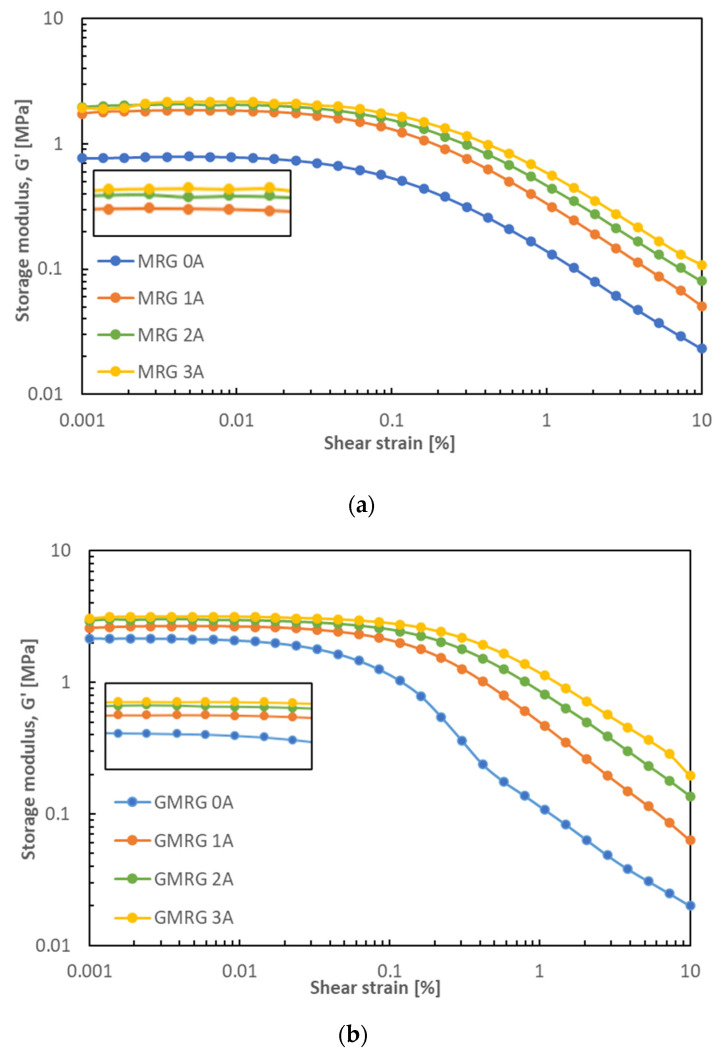
Storage modulus G′ as of function of shear strain for (**a**) MRG and (**b**) GMRG at different applied currents.

Figure 6 illustrates the storage modulus G′ of MRG and GMRG under the applied strain ranging from 0.001% to 10% under different applied currents.

At the beginning of the applied strain, the storage modulus G′ of both samples was independent of strain amplitude, which is known as the dynamic property of linear viscoelastic (LVE) material. The plateau region is also reflected by the degree of entanglement of the fibrous network in a grease medium [33]. Nonetheless, the effect of strain on the storage modulus showed a non-linear trend as strain amplitude increased due to the change in the dynamic properties at high strain [34,35]. This finding is related to the destruction of the microstructure of the samples resulting from the strong distance dependence of dipole–dipole interactions, which is known as the Payne effect [35,36]. Additionally, at this stage, the overall entanglement level of the fibrous network structure of the thickening agent that provides the basic skeleton structure of grease also decreased, as it was broken by the high shear strain, which eventually led the grease to flow [37,38]. Generally, it is crucial to identify the LVE region of a material for use in specific applications.

Figure 6a shows that the storage modulus G′ of MRG increased from 0.77 to 1.74 MPa when increasing the applied current from 0 to 1 A. The dramatic increase in storage modulus G′ was caused by the strong columnar chain structures formed under a stronger magnetic field. Compared with Figure 6b, the different gaps in storage modulus G′ between 0 and 1 A for the GMRG samples were lower compared to those of the MRG samples. The addition of graphite to the GMRG sample increased the stiffness of the medium due to its high surface contact area, which led to a stronger interaction between the grease medium and the CIPs. Concurrently, this would affect the mobility of the CIPs in the medium [17]. Nevertheless, we observed that the storage modulus G′ of each sample slightly increased with increasing magnetic field strength.

**Figure 7:**
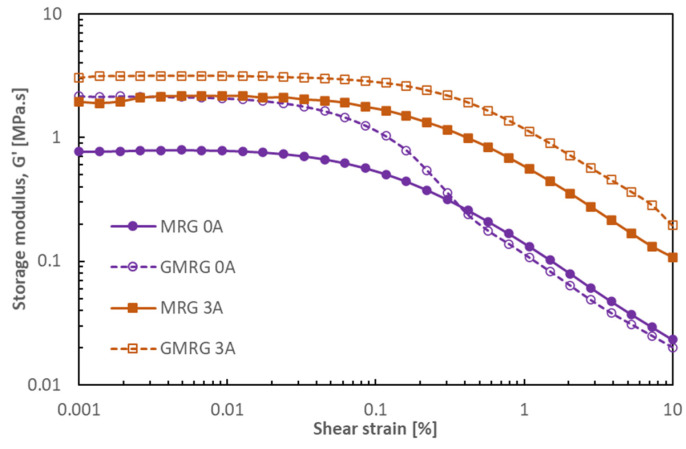
Storage modulus G′ as a function of shear strain for MRG and GMRG in the off- and on-state conditions.

Figure 7 illustrates the comparison of the MRG and GMRG samples under shear strain under off- and on-state conditions.

The storage modulus G′ of GMRG displayed a higher value compared to MRG at all magnetic field strengths. This finding reflects a strong viscoelastic behavior exhibited by the GMRG samples. The addition of graphite to the GMRG sample shortened the range of the LVE region in the off-state condition because graphite contributed to a stiffer GMRG that was sensitive to a low strain, which could cause microstructural damage. However, a broad LVE region (<0.1%) was observed in the GMRG sample compared to the MRG sample (<0.03%) in the on-state condition because the irregular shape of graphite contributed to good wettability between the graphite and grease, which led to an excellent dispersion of graphite in the grease medium [32]. As a result, graphite was also involved in the alignment process together with CIP, thus providing more a stable structure at high magnetic field strength.

**Figure 8:**
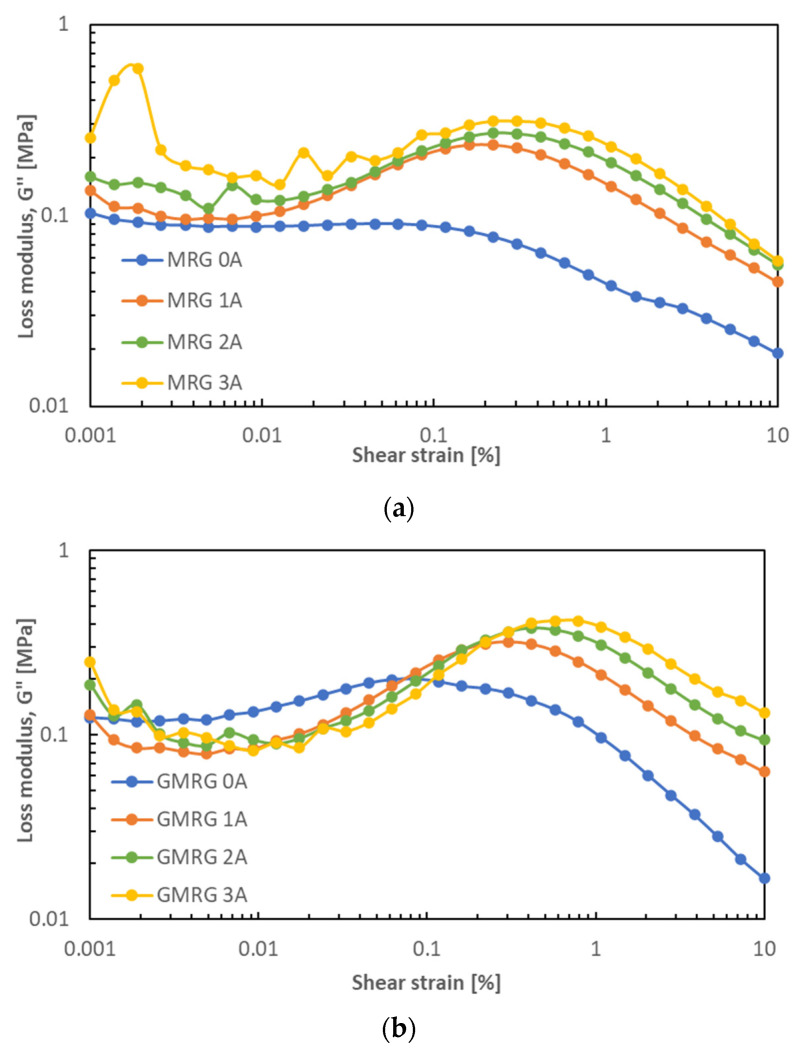
Loss modulus G″ of (**a**) MRG and (**b**) GMRG as a function of shear strain under different applied currents.

Figure 8 presents the loss modulus G″ of MRG and GMRG at small strain values ranging from 0.001% to 10% under different applied currents.

The loss modulus G″ for each sample showed a lower value at strain <0.1% but increased dramatically at >0.1%. Both samples displayed a fluctuating loss modulus at high magnetic field strengths and a low applied strain of <0.1%. Moreover, the peak of the graph became more evident with increasing magnetic field strength. This suggests that more energy dissipated through heat at high magnetic field strength, promoting more inter-particle interactions between CIPs [39]. However, the increasing loss modulus at a higher magnetic field might be due to the complicated structure attributed to the combination of the grease’s matrix fiber structure with an induced-magnetizable chain structure [40].

**Figure 9:**
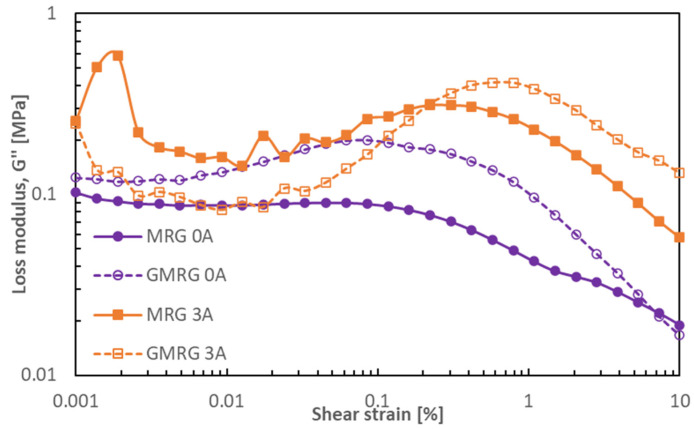
Loss modulus G″ as a function of shear strain for MRG and GMRG in the off- and on-state conditions.

The comparison of loss modulus G″ of both samples in the off- and on-state conditions is illustrated in Figure 9.

We observed that in the absence of a magnetic field, the loss modulus of GMRG was higher than that of MRG, indicating that more heat was dissipated by GMRG. In contrast, in the presence of a magnetic field, we noticed that the MRG sample displayed a higher and more fluctuating loss modulus compared to GMRG at a very low strain (<0.1%). A possible reason for this finding might relate to the stable structure acquired by GMRG at a higher applied magnetic field strength, caused by the existence of graphite particles. However, at a strain of >0.1%, GMRG showed a much higher loss modulus compared to MRG due to the strong Payne effect experienced by GMRG.

**Figure 10:**
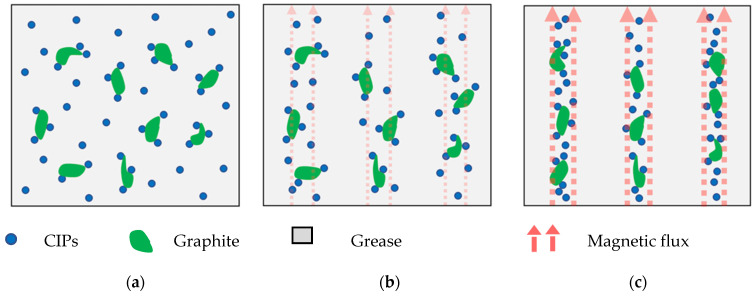
Schematic arrangement of CIPs and graphite in MRG: (**a**) in the absence of a magnetic field, (**b**) in the presence of a magnetic field, and (**c**) with further increases in the magnetic field.

Based on these results, it can be predicted that the addition of graphite has a significant effect on the interaction between CIPs and grease medium. We assumed that some of the CIPs were attracted to the graphite to establish bonding between them (Figure 1b). Furthermore, the improvement in rheological properties of GMRG was related to the excellent interfacial interaction of graphite with grease medium. The mechanism of the movement of graphite during CIP alignment in the presence of a magnetic field in GMRG is illustrated in Figure 10.

Without an application of a magnetic field, the spherical CIPs and irregular-shaped graphite were dispersed randomly [17] in the grease medium, as shown in Figure 10a. At this stage, the viscosity of the samples was primarily dependent on the fibrous structure of the grease matrix. Due to the good adhesion of graphite with the grease medium, the apparent viscosity and shear stress of GMRG appeared to be higher than those of MRG. However, the small difference compared to MRG was due to the low density of graphite.

Conversely, with the application of a magnetic field, the CIPs were magnetized and started to attract each other, as displayed in Figure 10b. The CIPs moved according to the direction of the magnetic field, and the gap between the particles simultaneously reduced. The alignment process involved the CIPs, where the graphite was also followed by the movement of CIPs attached to it. The inter-particle interactions were stronger with further increases in the magnetic field due to the smaller gap between the CIPs, resulting in a thick columnar chain structure produced from dipole–dipole forces. More CIPs accumulated around the graphite’s surface due to the rough and high surface area contact of irregular graphite [32]. Consequently, the graphite tended to “move” together with magnetizable CIPs and thus were involved in the formation of the columnar structure within the matrix, as shown in Figure 10c. Apparently, a stronger interaction structure between CIPs, graphite, and matrix led to the improvement in the rheological properties in the GMRG sample.

Compared to the previous additives in MRG, graphite improved the yield stress of MRG and expanded the range of yield stress further along with an increase in the magnetic field even though the off-state viscosity was observed to be slightly higher compared to MRG. Therefore, this new composition of MRG with graphite may be applied in many applications as it is convenient to handle and able to perform in a wide yield stress range.

## 4. Conclusions

A new type of MRG comprising 10 wt % graphite (GMRG) was prepared in this study, and its rheological properties were compared to those of MRG. The results showed that the GMRG sample had better rheological properties in terms of apparent viscosity, shear stress, yield stress, and viscoelastic properties. The GMRG demonstrated a better apparent viscosity in both off- and on-state conditions, reflecting the contribution of graphite filling the spaces between CIPs. Interestingly, the yield stress of GMRG improved by 38.8%, demonstrating the contribution of graphite to a stable structure. Furthermore, the addition of graphite in GMRG resulted in widening the yield stress from 0 to 3 A to about 32.7% higher than that of MRG. Additionally, GMRG presented a broader LVE region at 0.1% compared to MRG at 0.01% in 3 A, which produced an improvement in the elastic properties of GMRG through addition of graphite. These findings were further confirmed by the ESEM characterization of GMRG, which showed how graphite is involved in the formation of a columnar chain structure between the CIPs. Thus, our findings show how the addition of graphite as a carbon-based additive alters and improves the microstructure by strengthening the CIP columnar structure to enhance GMRG’s properties.

## Figures and Tables

**Table 1:** The composition of MRG and GMRG samples.

Samples	Percentage by Weight (wt %)		
	Grease	CIPs	Graphite
MRG	30	70	0
GMRG	20	70	10

**Table 2:** GMRG elemental composition.

Element	Weight (%)	Atomic (%)
**C**	75.34	89.26
**O**	7.02	6.24
**Fe**	17.64	4.49

## Data Availability

The raw/processed data required to reproduce these findings cannot be shared at this time as the data also part of an ongoing study.

## References

[1] Ahamed R., Choi S.B., Ferdaus M.M. (2018). A State of Art on Magneto-Rheological Materials and Their Potential Applications. J. Intell. Mater. Syst. Struct..

[2] Kumar J.S., Paul P.S., Raghunathan G., Alex D.G. (2019). A Review of Challenges and Solutions in the Preparation and Use of Magnetorheological Fluids. Int. J. Mech. Mater. Eng..

[3] Park J.H., Kwon M.H., Park O.O. (2001). Rheological Properties and Stability of Magnetorheological Fluids Using Viscoelastic Medium and Nanoadditives. Korean J. Chem. Eng..

[4] Rankin P.J., Horvath A.T., Klingenberg D.J. (1999). Magnetorheology in Viscoplastic Media. Rheol. Acta.

[5] Karis T.E., Kono R.N., Jhon M.S. (2003). Harmonic Analysis in Grease Rheology. J. Appl. Polym. Sci..

[6] Wang H., Li Y., Zhang G., Wang J. (2019). Effect of Temperature on Rheological Properties of Lithium-Based Magnetorheological Grease. Smart Mater. Struct..

[7] Mohamad N., Mazlan S.A., Ubaidillah, Choi S.B., Nordin M.F.M. (2016). The Field-Dependent Rheological Properties of Magnetorheological Grease Based on Carbonyl-Iron-Particles. Smart Mater. Struct..

[8] Burhannuddin N.L., Nordin N.A., Mazlan S.A., Aziz S.A.A., Kuwano N., Jamari S.K.M., Ubaidillah (2021). Physicochemical Characterization and Rheological Properties of Magnetic Elastomers Containing Different Shapes of Corroded Carbonyl Iron Particles. Sci. Rep..

[9] Sahin H., Gordaninejad F., Wang X., Fuchs A. (2007). Rheological Behavior of Magneto-Rheological Grease (MRG). SPIE 6525, Active and Passive Smart Structures and Integrated Systems.

[10] Sukhwani V.K., Hirani H. (2008). A Comparative Study of Magnetorheological-Fluid-Brake and Magnetorheological-Grease-Brake. Tribol. Online.

[11] Kim J.E., Ko J.D., Liu Y.D., Kim I.G., Choi H.J. (2012). Effect of Medium Oil on Magnetorheology of Soft Carbonyl Iron Particles. IEEE Trans. Magn..

[12] Mohamad N., Rosli M.A., Abdul Aziz S.A., Mazlan S.A., Ubaidillah U., Nordin N.A., Yahaya H., Abd Fatah A.Y., Sabino U., Imaduddin F., Prabowo A. (2020). Intrinsic Apparent Viscosity and Rheological Properties of Magnetorheological Grease with Dilution Oils. Lecture Notes in Mechanical Engineering, Proceedings of the 6th International Conference and Exhibition on Sustainable Energy and Advanced Materials, Surakarta, Indonesia, 16–17 October 2019.

[13] Wang K., Dong X., Li J., Shi K., Li K. (2019). Effects of Silicone Oil Viscosity and Carbonyl Iron Particleweight Fraction and Size on Yield Stress for Magnetorheological Grease Based on a New Preparation Technique. Materials.

[14] Mohamad N., Ubaidillah, Mazlan S.A., Choi S.B., Halim N.A. (2018). Improvement of Magnetorheological Greases with Superparamagnetic Nanoparticles. MATEC Web Conf..

[15] Tarmizi S.M.A., Nordin N.A., Mazlan S.A., Mohamad N., Rahman H.A., Aziz S.A.A., Nazmi N., Azmi M.A. (2020). Incorporation of Cobalt Ferrite on the Field Dependent Performances of Magnetorheological Grease. J. Mater. Res. Technol..

[16] Tian T.F., Li W.H., Alici G., Du H., Deng Y.M. (2011). Microstructure and Magnetorheology of Graphite-Based MR Elastomers. Rheol. Acta.

[17] Shabdin M.K., Rahman M.A.A., Mazlan S.A., Ubaidillah, Hapipi N.M., Adiputra D., Aziz S.A.A., Bahiuddin I., Choi S.B. (2019). Material Characterizations of Gr-Based Magnetorheological Elastomer for Possible Sensor Applications: Rheological and Resistivity Properties. Materials.

[18] Pang H., Xuan S., Liu T., Gong X. (2015). Magnetic Field Dependent Electro-Conductivity of the Graphite Doped Magnetorheological Plastomers. Soft Matter.

[19] Thakur M.K. (2020). Influence of Graphite Flakes on the Strength of Magnetorheological Fluids at High Temperature and Its Rheology. IEEE Trans. Magn..

[20] Radouane N., Maaroufi A., Ouaki B., Poupin C., Cousin R., Duponchel B., Singh D.P., Hadj-Sahraoui A., Depriester M. (2020). Thermal, Electrical and Structural Characterization of Zinc Phosphate Glass Matrix Loaded with Different Volume Fractions of the Graphite Particles. J. Non. Cryst. Solids.

[21] Sengupta R., Bhattacharya M., Bandyopadhyay S., Bhowmick A.K. (2011). A Review on the Mechanical and Electrical Properties of Graphite and Modified Graphite Reinforced Polymer Composites. Prog. Polym. Sci..

[22] Porfir’ev Y.V., Popov P.S., Zaichenko V.A., Shavalov S.A., Kotelev M.S., Kolybel’skii D.S., Tonkonogov B.P. (2019). Effect of Thickeners on Low-Temperature Greases. Chem. Technol. Fuels Oils.

[23] Paszkowski M., Olsztyńska-Janus S., Wilk I. (2014). Studies of the Kinetics of Lithium Grease Microstructure Regeneration by Means of Dynamic Oscillatory Rheological Tests and FTIR-ATR Spectroscopy. Tribol. Lett..

[24] Park B.O., Park B.J., Hato M.J., Choi H.J. (2011). Soft Magnetic Carbonyl Iron Microsphere Dispersed in Grease and Its Rheological Characteristics under Magnetic Field. Colloid Polym. Sci..

[25] Kavlicoglu B.M., Gordaninejad F., Wang X. (2013). Study of a Magnetorheological Grease Clutch. Smart Mater. Struct..

[26] Wang X., Gordaninejad F. (2006). Study of Magnetorheological Fluids at High Shear Rates. Rheol. Acta.

[27] Czarny R., Paszkowski M. (2007). The Influence of Graphite Solid Additives, MoS2 and PTFE on Changes in Shear Stress Values in Lubricating Greases. J. Synth. Lubr..

[28] Khan S.A., Lazoglu I. (2019). Development of Additively Manufacturable and Electrically Conductive Graphite—Polymer Composites. Prog. Addit. Manuf..

[29] Zhang W.L., Kim S.D., Choi H.J. (2014). Effect of Graphene Oxide on Carbonyl-Iron-Based Magnetorheological Fluid. IEEE Trans. Magn..

[30] Prasad M.H., Gangadharan K.V. (2014). Synthesis and Magneto Mechanical Properties of MR Grease. Int. J. Eng. Res. Technol..

[31] Tian Y., Jiang J., Meng Y., Wen S. (1993). A Shear Thickening Phenomenon in Magnetic Field Controlled-Dipolar Suspensions. IEEE Trans. Inf. Theory.

[32] Baptista R., Mendão A., Rodrigues F., Figueiredo-Pina C.G., Guedes M., Marat-Mendes R. (2016). Effect of High Graphite Filler Contents on the Mechanical and Tribological Failure Behavior of Epoxy Matrix Composites. Theor. Appl. Fract. Mech..

[33] Delgado M.A., Franco J.M., Kuhn E. (2008). Effect of Rheological Behaviour of Lithium Greases on the Friction Process. Ind. Lubr. Tribol..

[34] Ubaidillah B., Sutrisno J., Purwanto A., Mazlan S.A. (2015). Recent Progress on Magnetorheological Solids: Materials, Fabrication, Testing, and Applications. Adv. Eng. Mater..

[35] Xu Y., Gong X., Xuan S. (2013). Soft Magnetorheological Polymer Gels with Controllable Rheological Properties. Smart Mater. Struct..

[36] Gong X., Xu Y., Xuan S., Guo C., Zong L. (2012). The Investigation on the Nonlinearity of Plasticine-like Magnetorheological Material under Oscillatory Shear Rheometry. J. Rheol..

[37] Delgado M.A., Valencia C., Sánchez M.C., Franco J.M., Gallegos C. (2006). Thermorheological Behaviour of a Lithium Lubricating Grease. Tribol. Lett..

[38] Sánchez M.C., Franco J.M., Valencia C., Gallegos C., Urquiola F., Urchegui R. (2011). Atomic Force Microscopy and Thermo-Rheological Characterisation of Lubricating Greases. Tribol. Lett..

[39] Helgeson M.E., Wagner N.J., Vlassopoulos D. (2007). Viscoelasticity and Shear Melting of Colloidal Star Polymer Glasses Viscoelasticity and Shear Melting of Colloidal Star Polymer Glasses. J. Rheol..

[40] Wang H., Chang T., Li Y., Li S., Zhang G., Wang J., Li J. (2020). Characterization of Nonlinear Viscoelasticity of Magnetorheological Grease under Large Oscillatory Shear by Using Fourier Transform-Chebyshev Analysis. J. Intell. Mater. Syst. Struct..

